# New Books

**Published:** 2006-02

**A Field Guide for Science Writers: The Official Guide of the National Association of Science Writers, 2nd Ed.**

Deborah Blum, Mary Knudson, Robin Marantz Henig, eds.

New York:Oxford University Press, 2005. 368 pp. ISBN: 0-19-517498-4, $54.50

**Bioterrorism Preparedness: Medicine, Public Health, Policy**

Nancy Khardori

Hoboken, NJ:John Wiley & Sons, Inc., 2006. 276 pp. ISBN: 3-527-31235-8, $165

**Coal Combustion Byproducts and Environmental Issues**

K.S. Sajwan, I. Twardowska, T. Punshon, A.K. Alva, eds.

New York:Springer, 2006. 242 pp. ISBN: 0-387-25865-5, $169

**Ecological Literacy: Educating Our Children for a Sustainable World**

Michael K. Stone, Zenobia Barlow, eds.

San Francisco, CA:Sierra Club, 2005. 256 pp. ISBN: 1-57805-153-3, $16.95

**Fed Up! Winning the War Against Childhood Obesity**

Susan Okie

Washington, DC:Joseph Henry Press, 2005. 322 pp. ISBN: 0-309-09310-4, $27.95

**Genome Instability in Cancer Development**

Erich A. Nigg, ed.

New York:Springer, 2005. 511 pp. ISBN: 1-4020-3763-5, $159

**Global Change and Mountain Regions: An Overview of Current Knowledge**

Uli M. Huber, Harald K.M. Bugmann, Mel A. Reasoner, eds.

New York:Springer, 2005. 650 pp. ISBN: 1-4020-3507-1, $99

**Inhalation Toxicology, 2nd Ed.**

Harry Salem, Sidney A. Katz

Boca Raton, FL:CRC Press, 2005. 1056 pp. ISBN: 0-8493-4049-7, $189.95

**Mercury Hazards to Living Organisms**

Ronald Eisler

Boca Raton, FL:CRC Press, 2006. 328 pp. ISBN: 0-8493-9212-8, $169.95

**Metabolomics**

M. Tomita, T. Nishioka, eds.

New York:Springer, 2005. 256 pp. ISBN: 4-431-25121-9, $99

**Mitigation of Natural Hazards and Disasters: International Perspectives**

C. Emdad Haque, ed.

New York:Springer, 2005. 240 pp. ISBN: 1-4020-3112-2, $99

**Nanochemistry: A Chemical Approach to Nanomaterials**

Geoff Ozin, A. Arsenault

New York:Springer, 2005. 594 pp. ISBN: 0-85404-664-X, $89.95

**Ontologies for Bioinformatics**

Kenneth Baclawski, Tianhua Niu

Cambridge, MA:MIT Press, 2005. 440 pp. ISBN: 0-262-02591-4, $45

**Precautionary Tools for Reshaping Environmental Policy**

Nancy J. Myers, Carolyn Raffensperger, eds.

Cambridge, MA:MIT Press, 2005. 400 pp. ISBN: 0-262-63323-X, $25

**Principles of Ecotoxicology, 3rd Ed.**

C.H. Walker, S.P. Hopkin, R.M. Sibly, D.B. Peakall

Boca Raton, FL:CRC Press, 2005. 344 pp. ISBN: 0-8493-3635-X, $69.95

**The Global Genome: Biotechnology, Politics, and Culture**

Eugene Thacker

Cambridge, MA:MIT Press, 2005. 464 pp. ISBN: 0-262-20155-0, $39.95

**The Poison Paradox: Chemicals As Friends and Foes**

John Timbrell

New York:Oxford University Press, 2005. 368 pp. ISBN: 0-19-280495-2, $29.95

**The Quest for Environmental Justice: Human Rights and the Politics of Pollution**

Robert D. Bullard, ed.

San Francisco, CA:Sierra Club, 2005. 384 pp. ISBN: 1578051207, $18.95

**Toxicogenomic Technologies and Risk Assessment of Environmental Carcinogens: A Workshop Summary**

Committee on How Toxicogenomics Could Inform Critical Issues in Carcinogenic Risk Assessment of Environmental Chemicals, Committee on Emerging Issues and Data on Environmental Contaminants, National Research Council

Washington, DC:National Academies Press, 2005. 68 pp. ISBN: 0-309-09700-2, $10.80

**Toxicology of the Lung, 4th Ed.**

Donald E. Gardner

Boca Raton, FL:CRC Press, 2005. 696 pp. ISBN: 0-8493-2835-7, $159.95

